# Observation of a topologically protected state in a magnetic domain wall stabilized by a ferromagnetic chemical barrier

**DOI:** 10.1038/s41598-018-35039-6

**Published:** 2018-11-12

**Authors:** Sandra Ruiz-Gómez, Michael Foerster, Lucia Aballe, Mariana P. Proenca, Irene Lucas, José Luis Prieto, Arantzazu Mascaraque, Juan de la Figuera, Adrián Quesada, Lucas Pérez

**Affiliations:** 1 Department Física de Materiales, Universidad Complutense de Madrid, 28040 Madrid, Spain; 2 Alba Synchrotron Light Facility, CELLS, E-08280 Bellaterra, Spain; 3 IFIMUP and IN - Institute of Nanoscience and Nanotechnology and Department Física e Astronomia, Universidad Porto, Rua do Campo Alegre 687, 4169-007 Porto, Portugal; 4 Instituto de Sistemas Optoelectrónicos y Microtecnología (ISOM), Universidad Politécnica de Madrid, Avda. Complutense 30, 28040 Madrid, Spain; 5 Dpto. Física de la Materia Condensada, Universidad de Zaragoza, Pedro Cerbuna 12, 50009 Zaragoza, Spain; 6 Instituto de Nanociencia de Aragón (INA), Universidad de Zaragoza, Mariano Esquillor, Edificio I+D, 50018 Zaragoza, Spain; 7 Unidad Asociada IQFR (CSIC)-UCM, 28040 Madrid, Spain; 8 Instituto de Química Física “Rocasolano” CSIC, 28006 Madrid, Spain; 9 Instituto de Cerámica y Vidrio, ICV-CSIC, 28049 Madrid, Spain; 10 IMDEA Nanociencia, 28049 Madrid, Spain

## Abstract

The precise control and stabilization of magnetic domain walls is key for the development of the next generation magnetic nano-devices. Among the multitude of magnetic configurations of a magnetic domain wall, topologically protected states are of particular interest due to their intrinsic stability. In this work, using XMCD-PEEM, we have observed a topologically protected magnetic domain wall in a ferromagnetic cylindrical nanowire. Its structure is stabilized by periodic sharp alterations of the chemical composition in the nanowire. The large stability of this topologically protected domain wall contrasts with the mobility of other non-protected and non-chiral states also present in the same nanowire. The micromagnetic simulations show the structure and the conditions required to find the topologically protected state. These results are relevant for the design of future spintronic devices such as domain wall based RF oscillators or magnetic memories.

## Introduction

The possibility of manipulating local magnetization by spin polarized currents^1^ or pure spin currents^2^ is one of the most relevant advances in modern condensed matter physics. Among the multitude of possibilities arising from the control of spin currents, the interaction of these currents with magnetic domain walls is of particular importance. The next generation of magnetic storage technology may well be based on domain walls (DWs) manipulated by spin-polarized currents^3–5^. Other important proposals make use of DWs for RF generation^6,7^, energy storage^8^ or even for spin wave channeled transmission^9^.

In any of these applications, the DW has to be stabilized at a particular point in the device in a controlled and reliable manner. Geometrical constrictions^10^ have been widely used in recent years but they promote non-uniform distribution of the spin-polarized current and a sizable Joule heating associated to the local increase of current density^11^. Instead, a much more optimized strategy is to use structural, chemical or topological defects^12,13^ to stabilize the DW or any other spin structure such a skyrmion^14^. Some examples include the use of localized ion bombardment^15^, the application of local electric and magnetic fields^10,16^ or the introduction of a sharp change in the chemical composition in the structure^17,18^. Although this last option, a sharp change in the chemical composition, seems to be a promising and reliable solution to stop a DW at a particular point of the nanodevice, it cannot be easily implemented in ferromagnetic nanostripes. On the other hand, a sharp change in chemical composition can be readily engineered by electrodeposition in a cylindrical nanowire^19^, a geometry with added advantages such as the possibility of having energetically stable chiral spin structures^20^. Interestingly, these chiral DWs could be topologically protected under specific conditions although, to our knowledge and despite the technological relevance of this DW state, an experimental observation of this protected state has not been reported so far.

In this work we show the first experimental evidence of the stabilization of a topologically protected state of a domain wall by a change of composition in the ferromagnetic material. In electrodeposited ferromagnetic nanowires, we have introduced periodic compositional ferromagnetic changes — chemical barriers — that act as a pinning site able to stabilize two different magnetic domain wall structures: one topologically protected and a non-chiral DW that can be moved reversibly with a moderated external magnetic field. The experimental characterization of both DW structures by XMCD-PEEM is supported by micromagnetic simulations. Our results provide an attractive pathway for the fabrication of optimized nano-devices based on the manipulation of DWs with spin-polarized currents.

**Figure 1:**
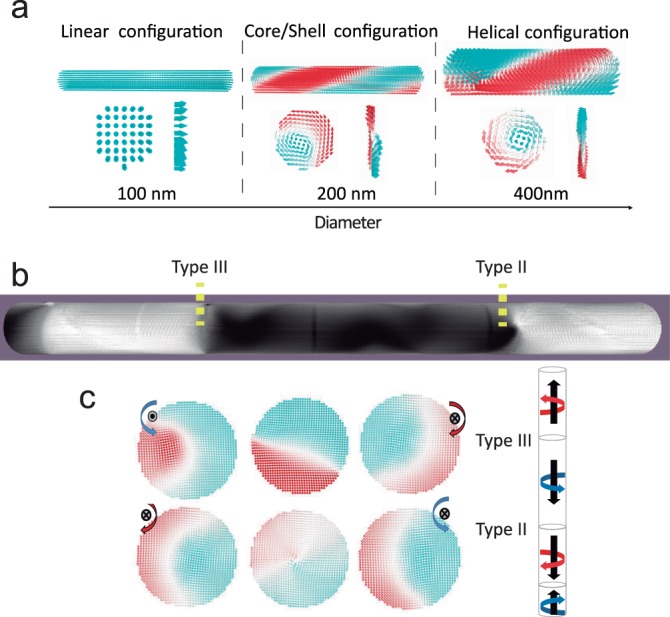
Micromagnetic simulation of the evolution of magnetic configuration for NWs with different diameters. (**b**) Shows the magnetization state of minimum energy for zero external magnetic field for a wire with chemical notches (yellow dash line). (**c**) Upper row (lower row) shows the cross-section of the magnetic configuration in a type III (type II) DW in the position of the wall (central column), and just before and after the DW. The color scale represents the component along the axis (blue and red) and perpendicular to the axis (black and white).

**Figure 2:**
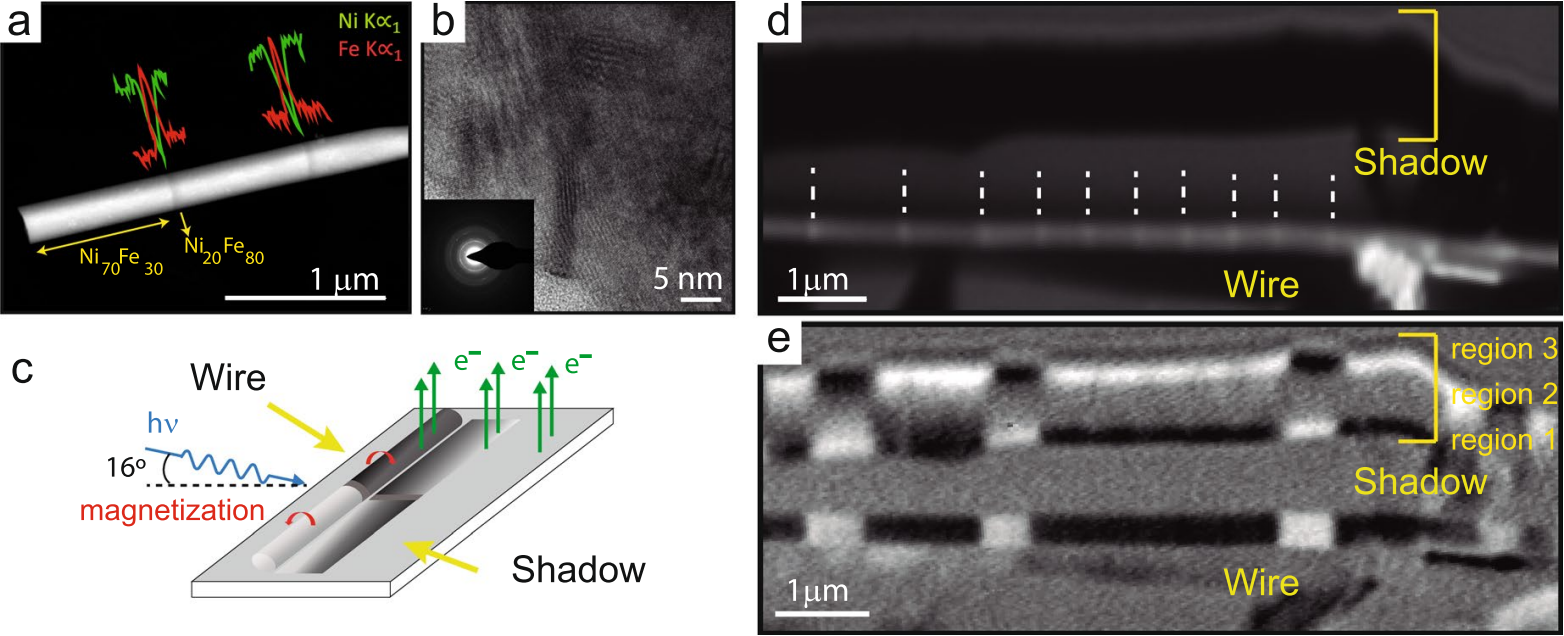
HR-TEM images of a single nanowire with chemical notches. (**a**) Z-contrast image, together with a compositional profile measured by EDX along the nanowire. Red line corresponds to the Fe content and green line to the Ni content. (**b**) HR-TEM image in which the polycrystalline character of the nanowire can be observed. The inset of panel b shows a SAED pattern measured in the nanowire. (**c**) Schematics of the measurement configuration, (**d**) chemical contrast image for a wire with chemical notches separated by 800 nm and (**e**) XMCD image at FeL_3_ edge for the same wire, where it is possible to distinguish magnetic contrast in the wire and also in the shadow of the wire, with three regions marked as 1, 2 and 3.

Electrodeposition was employed in this work to fabricate FeNi nanowires (NWs) with equidistant changes in composition (chemical barriers) along their length. Experimentally, the composition of electrodeposited Fe-Ni NWs can be controlled during growth by changing the applied potential^21^. The choice of diameter for the NWs is determined by the sizes that allow chiral magnetization configurations which, for Permalloy nanowires, happens for diameters close to and above 200 nm, as shown by micromagnetic simulations (see Fig. 1a). We have grown nanowires with a diameter of 200 nm by pulsing the applied voltage between V_1_ = −1.5 V and V_2_ = −1.0 V (vs. Ag/AgCl). During the first pulse, a homogeneous NW segment is grown, whose length was varied between ~250 nm and 1000 nm by tuning the electrodeposition time. The second pulse introduces a short region (chemical barrier) in which the composition changes, increasing the Fe content in the alloy. With this procedure, we grow a NW homogeneous in diameter with a uniform Fe_30_Ni_70_ composition except in some periodic short regions where the composition changes to Fe_80_Ni_20_. One of these NWs is imaged with TEM in Z-contrast mode in Fig. 2a. As shown in the image, the chemical barriers are approximately 20 nm wide and they are separated 900 nm from each other. The composition profiles along different NWs measured by energy dispersive X-ray spectroscopy (EDX), show sharp changes in the Fe/Ni ratio at the chemical barriers. The composition change from Fe_30_Ni_70_ to Fe_80_Ni_20_ implies a variation of the saturation magnetization from *μ*_0_M_S_ = 0.8 T to *μ*_0_M_S_ = 1.4 T respectively.

High resolution TEM images (Fig. 2b) show that the wires are polycrystalline with a small crystalline grain size, as visible in the selected area electron diffraction (SAED) pattern (inset to Fig. 2b). The disorder in the crystalline structure is related to the large overpotential used to grow the NWs and it is particularly important in the NWs under study. Due to the disorder, the magnetocrystalline anisotropy is randomly oriented along the nanowire and therefore the magnetization dynamics are controlled mainly by the overall shape anisotropy and by the magnetic poles induced in the chemical barriers due to the change in saturation magnetization.

In order to study the correlation between chemical composition and magnetic properties at the nanometer scale it is necessary to use a microscopy technique that combines chemical and magnetic imaging capabilities. In this work we have used a combination of photoemission electron microscopy (PEEM) with X-ray absorption (XAS) and X-ray magnetic circular dichroism (XMCD) spectroscopies^22^. In particular, we have measured XAS images at the FeL_3_ and NiL_3_ absorption edges (707.2 and 851.8 eV, respectively), with both right-handed and left-handed X-ray polarization, and with the incident beam forming an angle of 16° with the surface (see Fig. 2c).

An image with chemical contrast can be obtained by subtracting XAS-PEEM images measured at the FeL_3_ and NiL_3_ absorption edges, as shown in Fig. 2d. This image highlights the Fe-rich chemical barriers as equi-spaced brighter zones. Additionally, magnetic images can be obtained by XMCD-PEEM, as the difference between the PEEM images measured at the FeL_3_ absorption edges with positive and negative polarization. In Fig. 2e the XMCD-PEEM image is shown after demagnetizing the sample in an alternating magnetic field. The white and black contrast in XMCD-PEEM images corresponds to areas in which the magnetization lies along or opposite to the incident x-ray beam direction. In Fig. 2e the direction of the incident photon beam was nearly perpendicular to the NW axis, pointing upwards in the image. There are two different areas which show magnetic contrast: the wire and its shadow. In the first case, the magnetic contrast arises from electrons coming from the surface of the NW (~5 nm in depth). In the second case (the shadow) the magnetic contrast area corresponds to electrons photoemitted from the substrate, excited by the X-ray beam transmitted through the wire^23–25^ (see also the schematic drawing in Fig. 2c). Therefore, by analysing the magnetic contrast in the shadow area, we can obtain information of the magnetic configuration in the bulk of the nanowire while the wire gives mostly surface information. The contrast in the region 3 of the shadow corresponds to the beam transmitted through the upper part of the wire. The magnetic information should be the same as the one seen in the nanowire but with opposite magnetic contrast, since the absorbed and transmitted X-rays are complementary. Region 1 corresponds to the bottom part of the wire. The magnetic contrast of this region is the opposite to the one observed in region 3, indicating that the upper and the bottom part of the nanowire are magnetized in opposite directions. This indicates a helical magnetic configuration. In fact, in region 2, which is an image created by photons transmitted through the middle of the nanowire, there is no visible contrast and there is an abrupt separation of grey and black/white. Therefore the magnetization direction in the bulk of the nanowire lies along its axis (normal to the beam). Thus the wire has a core-shell structure with core magnetized along the axis of the wire and a helical magnetic configuration in the shell, as expected from the micromagnetic simulations shown in Fig. 1.

Comparing the XAS-PEEM (Fig. 2d) and the XMCD-PEEM (Fig. 2e) images, the location of the chemical barriers can be correlated with the magnetic configuration. Virtually all the observed magnetic domain walls are located at the areas where there is a change in the composition of the NW. For the 64 DWs observed, in wires with chemical barriers separated 250 nm, 400 nm and 900 nm, 95% of those DWs are located at the chemical barriers. Therefore, the chemical barriers are very effective pinning sites for the magnetic DWs.

Micromagnetic simulations have been performed in nanowires with a fixed length of 6 *μ*m. The chemical barriers have been introduced in the nanowires as a change in saturation magnetization in selected and equi-spaced areas and they are marked with yellow dashed lines in Fig. 1. Figure 1b shows the magnetization state of minimum energy for zero external magnetic field for a wire with chemical barriers. All the DWs find their resting position at the chemical barriers.

**Figure 3:**
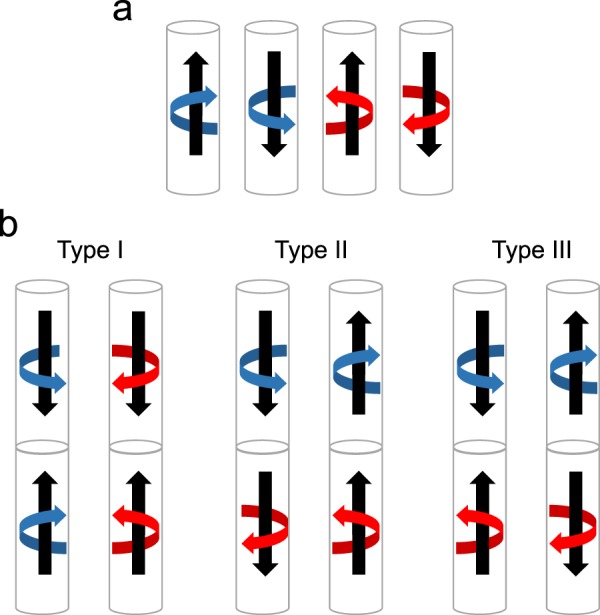
(**a**) Scheme of the four different magnetic domains and (**b**) six possible domains walls obtained combining all the magnetic domains.

The DWs displayed in Fig. 1 can separate domains with four possible configurations as shown in Fig. 3a, depending on the core magnetization direction (upwards or downwards) and on the shell chirality (clockwise and counter-clockwise) and two with the projection in the opposite direction, rotating clockwise and counter- clockwise. These four domain configuration lead to six possible different domains walls separating them, although they can be classified in three types, as shown schematically in Fig. 3b. In Type I, the shell chirality is conserved across the DW and only the direction of the core magnetization is switched. Type I DW cannot be distinguished by XMCD-PEEM when illuminating the sample perpendicular to the wire as the component of the magnetization parallel to the beam direction is the same for both domains. On the other hand, Type II DW keeps the direction of magnetization in the core, but it separates opposite chirality in the shell. In this case, when illuminating the sample perpendicular to the wire by XMCD-PEEM, the component of the magnetization parallel to the beam is opposite for both domains and, therefore Type II DW can be detected experimentally with our geometry. The detailed structure of Type II domain wall, obtained by micromagnetic simulation, is shown in 1c. This type of domain wall is topologically protected^26^ and it is very difficult to move under the application of a longitudinal magnetic field. Finally, there is a third type of DW, Type III, which separate domains with opposite core magnetization and opposite shell chirality. Type III can also be observed by XMCD-PEEM because, as for Type II, the component of the magnetization parallel to the beam direction is opposite for both domains. The detailed structure of Type III DW, obtained by micromagnetic simulations, is shown in Fig. 1c.

**Figure 4:**
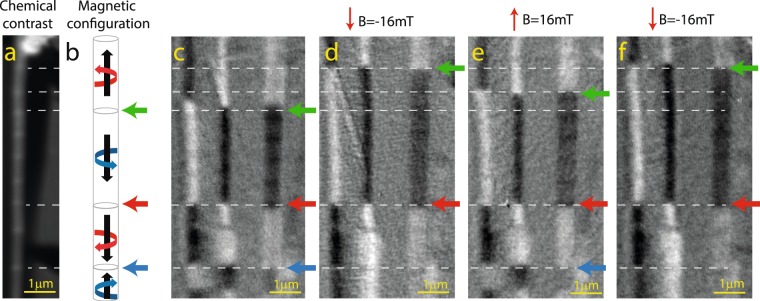
(**a**) Chemical contrast image for a wire with chemical notches separated by 800 nm. (**b**) Magnetic configuration of the nanowire. XMCD image at FeL_3_ edge for the same wire before (**c**) and after applying a magnetic field sequence of (**d**) −16 mT, (**e**) +16 mT and (**f**) −16 mT.

In order to test the resilience of the Type II topologically protected state we recorded XMCD images after the application of 1 s magnetic field pulses of various amplitudes, with direction parallel to the NW axis. The data collected from one of these experiments, using the same wire as in Fig. 2 (after being demagnetized) are summarized in Fig. 4. The chemical contrast image (Fig. 4a), shows the position of some of the chemical barriers, marked with horizontal dashed lines. In the initial state, all DWs are pinned in one of the chemical barriers. We consider first the DW located in the position marked by a green arrow in Fig. 4c. After applying a downwards pulse of B = 16 mT along the vertical direction of the NW, the DW jumps two barriers up (Fig. 4d). By applying the same magnetic field pulse in the opposite direction, the DW movement can be reversed (Fig. 4e). This process can be repeated so, by applying another downwards pulse, the DW can jump upwards again (Fig. 4f). A similar behavior but with opposite travelling directions is found in the DW marked with the blue arrow. From the observed motion of the DWs we can deduce that the DWs marked with green and blue arrows separate domains with opposite axial magnetization. This implies that the DW marked with a red arrow separates domains with the same axial magnetization but opposite shell chirality. This is a Type II DW, topologically protected and therefore insensitive to the magnetic field sequence, as shown in figures Fig. 4c to f. Type II DW cannot be moved with an external magnetic field even when pinned at the same chemical defect where a non-protected type III could be easily depinned (see Supplementary Information). This reinforces the fact that type II is very insensitive to an external magnetic field because it is a topologically protected configuration rather than because it is pinned at a very strong structural defect.

A total of 75 Type III DW movements have been followed in wires with distances between chemical notches ranging from 250 nm to 900 nm. The magnetic field component along the NW needed for the deppining of the DWs is 19 ± 2 mT for 250 nm, 12 ± 1 mT for 400 nm and 15 ± 1 mT for 900 nm. We were not able to move Type II DW in any of the experiments, even applying the largest field available in the experiment (160 mT).

To summarize, our results demonstrate that chemical boundaries in the magnetic nanowires can stabilize topologically protected DW states. Chemical discontinuities show good potential to stop topologically protected DWs at a given point in the nanodevice. This could be very useful for instance when designing DW-based RF oscillators. In these type of devices the domain wall oscillates around its equilibrium position, excited by a spin-polarized current. Even if the device requires the use of a large current density for the DW oscillation, it is unlikely going to move irreversibly a topologically protected DW, giving the necessary stability to the device. Additionally, in other DW based devices such as magnetic memories, the use of very resilient topologically protected DWs is very relevant for the durability of the information stored. The concept of a chemical barrier is in principle applicable to any chiral spin texture including skyrmions^27^ and to any DW-based storage^28^, logic^29,30^ and sensing device in general^31^.

## Materials and Methods

We have electrodeposited arrays of permalloy NWs by electrochemical deposition using nanoporous alumina membranes with a diameter of 200 nm purchased from Whatman as templates. Electrodeposition was carried out in a three-electrode electrochemical cell in a Ecochemie Autolab PGSTAT potentiostat, using a Pt mesh as a counter electrode and a Ag/AgCl (3 M NaCl) electrode as a reference electrode. Before electrodeposition, a thin Au film was thermally evaporated on one side of the membrane to act as working electrode. Permalloy NWs were grown at room temperature by pulsed electrodeposition. The electrolyte used is composed of NiSO_4_ (0.8 M) and NiCl_2_ (0.02 M) as Ni^2+^ sources, FeSO_4_ (0.16 M) as Fe^2+^ source and H_3_BO_3_ (0.4 M) and saccharine (0.016 M) as additives. All chemicals were of analytical grade and they were used without further purification and mixed in deionized water. The pH was adjusted to 2.3 using H_2_SO_4_ 10% vol.

The morphology of the NWs and the composition along the length of individual NWs were measured with a transmission electron microscope (TEM) JEOL JEM 2000FX. Before measurement, the gold layer was removed using a 0.1 M I_2_ and 0.6 M KI solution, the alumina template was dissolved in 0.4 M H_3_PO_4_ and 0.2 M H_2_CrO_4_ solution and the wires were dispersed in EtOH 99.5% vol. Some drops of the resulted solution were placed on a TEM grid.

XMCD-PEEM measurements were performed to establish a relation between the magnetic configuration of the NWs and the periodicity of the chemical barriers. Field pulses were applied *in situ* using a sample holder equipped with a quadrupole capable of providing a maximum in-plane field of 30 mT/A^32^. The PEEM measurements were performed at the CIRCE beamline of the ALBA Synchrotron^22^. For XMCD-images FeL_3_-edge were taken with opposite photon helicity, and subtracted pixel by pixel in order to determine the in-plane magnetization component along the X-ray direction with nanometer resolution. Chemical contrast images were be obtained as the difference in X-ray absorption at the FeL_3_ and NiL_3_ absorption edges. In all cases images were acquired by measuring the distribution of secondary electrons at low kinetic energies.

Micromagnetic simulations were performed with the mumax3 code^33^. The total length of the cylindrical NWs was fixed to 6 µm and the diameter was set to 200 nm. The length of the Ni-rich layers was varied from 250 nm to 1000 nm and the length of the chemical barrier was kept constant at 20 nm. Finite element discretization size was chosen to be 4 nm for both layers. For both type of layers we consider a structure without magnetocrystalline anisotropy. The exchange constant was fixed at A_ex_ = 13 × 10^−12^ J/m and the damping constant at *α* = 0.02. The saturation magnetization was set to *μ*_0_M_S_ = 0.8 T in the NW and *μ*_0_M_S_ = 1.4 T in the chemical barriers.

## Electronic supplementary material

Supplementary Information
