# Supplementary material for: Observation of a topologically protected state in a magnetic domain wall stabilized by a ferromagnetic chemical barrier
Source: Sci Rep. 2018 Nov 12;8:16695. doi: 10.1038/s41598-018-35039-6 (PMC6232112; doi:10.1038/s41598-018-35039-6)
Supplement: Supplementary file 1 — Supplementary Information [file 41598_2018_35039_MOESM1_ESM.pdf]

Supplementary information:
Observation of a topologically protected state in a magnetic domain wall stabilized by a ferromagnetic chemical barrier

Sandra Ruiz-Gómez,[1] Michael Foerster,[2] Lucia Aballe,[2] Mariana P. Proenca,[3,4] Irene Lucas,[5,6] José Luis Prieto,[4] Arantzazu Mascaraque,[1,7] Juan de la Figuera,[8,7] Adrián Quesada,[9] and Lucas Pérez[1,7,10]

[1]Dept. Física de Materiales. Universidad Complutense de Madrid. 28040 Madrid, Spain
[2]Alba Synchrotron Light Facility, CELLS, E-08280 Bellaterra, Spain
[3]IFIMUP and IN - Institute of Nanoscience and Nanotechnology and Dep. Física e Astronomia, Univ. Porto, Rua do Campo Alegre 687, 4169-007 Porto, Portugal
[4]Instituto de Sistemas Optoelectrónicos y Microtecnología (ISOM), Universidad Politécnica de Madrid, Avda. Complutense 30, 28040 Madrid, Spain
[5]Dpto. Física de la Materia Condensada, Universidad de Zaragoza, Pedro Cerbuna 12, 50009 Zaragoza, Spain
[6]Instituto de Nanociencia de Aragón (INA), Universidad de Zaragoza, Mariano Esquillor, Edificio I+D, 50018 Zaragoza, Spain
[7]Unidad Asociada IQFR (CSIC)-UCM, 28040, Madrid, Spain
[8]Instituto de Química Física "Rocasolano" CSIC, 28006 Madrid, Spain
[9]Instituto de Cerámica y Vidrio, ICV-CSIC, 28049, Madrid, Spain
[10]IMDEA Nanociencia, 28049, Madrid, Spain

Pinning/depinning experiments of type II and type III domain walls.

A large pinning force could be caused by structural defects or areas in which nanowires are broken. To avoid these artefacts, we were very careful in selecting wires without broken or bended parts in our experiments. In order to demonstrate that type II domain walls are very insensitive to external magnetic field due to its topological nature and not because they are strongly pinned in defects, we carried out a domain walls motion experiment in which type II and type III domain walls (DWs) were pinned in the same chemical barrier. In the Figure S.1 we show a sequence of XMCD images taken in the Fe edge for a NW with chemical notches separated 250 nm. We represent type III walls with blue arrows and type II (the protected one) with red arrows. In the three first images there are two type II DWs that do not move under the application of a magnetic field and three type III DWs that can be easily moved. After demagnetizing the wire (with the largest available field in the system which is not able to move a type II wall), a new type II wall appears in the chemical barrier in which a type III wall was previously pinned and depinned. Once the type II DW is pinned in this chemical barrier, it cannot be moved by the magnetic field.

In this experiment we show that a type III DW can be pinned and depinned from this chemical barrier so there is no a structural effects but type II cannot be depinned from the same defect. This experiment clearly shows that type II DWs are topologically protected against the application of a magnetic field and not just trapped by structural defects or broken parts of a wire.

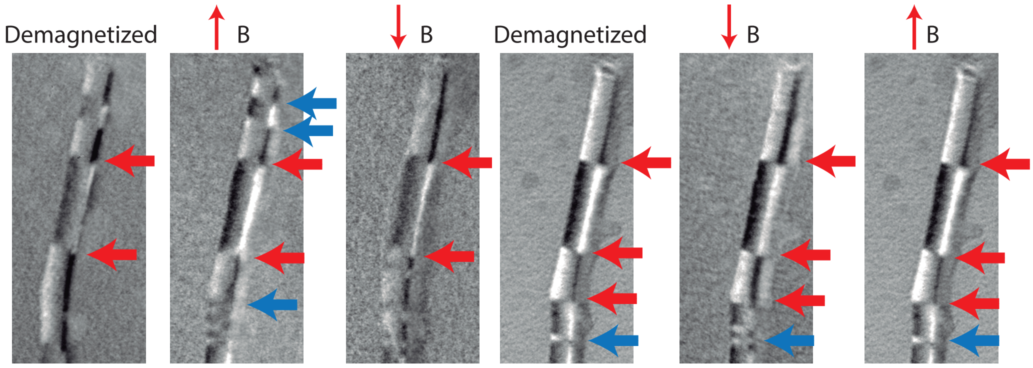

FIG. S.1: XMCD images for the same wire after a sequence consisting in: demagnetization (a), applying a magnetic field of 18mT (b) and -18mT (c), demagnetization again (d), applying a magnetic field of -18mT (e) and 18mT (f)
